# Enhanced Distributed Fiber Optic Vibration Sensing and Simultaneous Temperature Gradient Sensing Using Traditional C-OTDR and Structured Fiber with Scattering Dots

**DOI:** 10.3390/s19194114

**Published:** 2019-09-23

**Authors:** Konstantin Hicke, René Eisermann, Sebastian Chruscicki

**Affiliations:** 1 Bundesanstalt für Materialforschung und -prüfung (BAM), Unter den Eichen 87, 12205 Berlin, Germany; 2 Department High Voltage Engineering, Technische Universität Berlin (TUB), Einsteinufer 11, 10587 Berlin, Germany

**Keywords:** fiber optic sensors, distributed vibration sensing, DVS, distributed temperature gradient sensing, DTGS, C-OTDR, phase-sensitive OTDR, fiber structuring, fs-inscription

## Abstract

We present results demonstrating several beneficial effects on distributed fiber optic vibration sensing (DVS) functionality and performance resulting from utilizing standard single mode optical fiber (SMF) with femtosecond laser-inscribed equally-spaced simple scattering dots. This modification is particularly useful when using traditional single-wavelength amplitude-based coherent optical time domain reflectometry (C-OTDR) as sensing method. Local sensitivity is increased in quasi-distributed interferometric sensing zones which are formed by the fiber segments between subsequent pairs of the scattering dots. The otherwise nonlinear transfer function is overwritten with that of an ordinary two-beam interferometer. This linearizes the phase response to monotonous temperature variations. Furthermore, sensitivity fading is mitigated and the demodulation of low-frequency signals is enabled. The modification also allows for the quantitative determination of local temperature gradients directly from the C-OTDR intensity traces. The dots’ reflectivities and thus the induced attenuation can be tuned via the inscription process parameters. Our approach is a simple, robust and cost-effective way to gain these sensing improvements without the need for more sophisticated interrogator technology or more complex fiber structuring, e.g., based on ultra-weak FBG arrays. Our claims are substantiated by experimental evidence.

## 1. Introduction

Distributed acoustic sensing (DAS) or distributed vibration sensing (DVS) was developed more than 15 years ago and has since been used for a wide range of monitoring applications [1,2,3,4,5,6]. There is a large variety of sensing principles [7,8], most of which are based on the detection of Rayleigh backscatter from coherent optical pulses introduced into single mode optical fiber (SMF). This class of sensing schemes is called coherent optical time domain reflectometry (C-OTDR) or phase-sensitive OTDR (Φ-OTDR). The most simple approach to C-OTDR based DAS/DVS is based on the evaluation of varying backscatter intensity due to external perturbations [2,9,10,11]. However, this amplitude-based method does not allow to quantitatively determine vibration, or rather, dynamic strain amplitudes as it suffers from a highly nonlinear strain transfer function and signal fading [11,12,13,14]. Perturbations can only be detected rather than measured. Many DAS/DVS approaches have subsequently been developed to enable quantitative strain change measurements. The most common ones are based on the determination of the signal phase via phase demodulation or tracking [15,16,17,18,19,20,21]. Other state-of-the-art DAS/DVS techniques that allow for quantification of strain involve correlation analysis to ascertain shifts in Rayleigh backscatter profiles resulting from perturbations [22,23,24,25,26].

Many of the above-listed methods involve quite complex photonics on the sensing interrogator side, often with high demands on components like laser source, pulse shaper or detection path. In recent years, many investigations have been performed on the topic of how to improve C-OTDR DAS sensory performance from the side of the sensing fiber, proposing the use of modified optical fiber structured with large ultra-weak FBG arrays or continuous gratings [27,28,29,30,31,32,33,34,35,36,37,38,39,40]. Developments towards the demodulation of phase variations using discrete reflectors in optical fiber have, however, had a long history already. Many earlier works focused on mutiplexed interferometric sensing approaches, some also using FBGs, see e.g., [41,42,43,44,45]. Many of the developed methods were commercialized, e.g., [46] and utilized for i.a. industrial monitoring or subsea applications. Nowadays, continuous FBG arrays can be written into the sensing fiber online during the drawing process [47,48]. In recent works regarding DAS/DVS, the introduced FBGs took the role of strong and controllable reflectors in order to avoid the problem of the nonlinear, quasi-random strain transfer function. This method to improve phase-sensitive OTDR with a FBG-interferometric sensing scheme was first proposed by Zhu et al. [28]. The different proposed FBG array-enhanced DAS/DVS methods complement and work in conjunction with various phase demodulation approaches to C-OTDR (Φ-OTDR) in order to raise sensitivity, increase measurement precision or to attain greater sensing ranges by enhancing the backscatter [38,39,40].

However, FBG arrays used for distributed sensing exhibit unnecessary complexity and demand highly precise manufacturing processes which can increase costs. In previous work, our group outlined that inscribing simple strongly scattering dots (reflectors) via femtosecond laser pulses into the sensing fiber core (as was before demonstrated for polymer optical fiber [49,50]) also results in significant improvements of DVS with regard to sensitivity, fading and strain transfer regularization [51], which was also shown analogously for discrete UV-enhanced strong scattering segments [52]. Very recently Donko et al. proposed a similar technique with fs-inscribed discrete reflectors to enhance distributed sensing performance [53,54,55]. Fiber structuring based on scattering dots can be considered as simpler and has fewer demands than an FBG-based approach. FBGs are often written into a fiber using a UV laser and a phase mask, the fiber coating has then to be removed and the fiber recoated after the inscription, resulting in a reduced mechanical stability [48,56,57] whereas the fs-inscribed dots using a point-by-point technique [56,57,58] can be added without the need for uncoating, as the ultrashort pulses are launched to the fiber core and do not damage the coating. In order to manufacture large FBG arrays suitable for long range distributed sensing, online inscription techniques during the fiber drawing process like UV-laser based draw tower gratings [47,48] can be employed. This, however, adds to the complexity of and puts a high demand for vibrational stability on the draw tower installation to achieve high-quality gratings. Moreover, especially FBG arrays, even made of ultra-weak FBGs, increase the transmission losses significantly and therefore reduce the sensing length for DAS / DVS. In contrast to this, fs-laser writing is suitable to adjust the backscatter amplitude of point scatterers and write single point arrays over kilometer lengths in an industrial process. Furthermore, unlike inscibed dots, FBGs in arrays intended for use in distributed sensing with an interferometric approach need to match spectrally. In contrast to scattering dots, which function as broadband reflectors, draw tower FBGs often show inconsistencies with regard to their reflection spectra and are sensitive to environmental temperature changes [32,48]. On the other hand, utilizing large FBG arrays offers the possibility to combine or complement a distributed interferometric sensing approach with one making use of the high sensitivity and precision of FBG sensors when they are used as individual point sensors. While there are many studies describing the use of FBG arrays or continuous gratings for DAS/DVS, an in-depth investigation with regard to DAS/DVS performance using discrete strong scatterers has so far been lacking.

In this paper, we follow up on this and analyze the performance enhancements resulting from a sensing fiber structured by equally-spaced identical scatterers with strong reflectivity in the context of DVS based on amplitude-based single-wavelength direct-detection C-OTDR, which in the following we denote simply as (traditional) C-OTDR. Following parts of the scheme first proposed in [28], we show that the uniformly inscribed dots lead to a simplified transfer function and thus allow for the determination of monotonous phase changes on the period scale directly from the C-OTDR output intensity. This enables the quantification of transient perturbations like temperature changes or strain transients while the full sensing bandwidth which is only limited by the sensor length can still be exploited. We show that fiber segments between subsequent pairs of scattering dots form quasi-distributed two-beam interferometric sensing zones that, due to their extent, are less susceptible to noise-like thermal fluctuations. Sensitivity is then stabilized and the fading phenomenon thus mitigated. This together with the regularized transfer function also enables C-OTDR based quantitative distributed temperature gradient sensing (DTGS) simultaneous to distributed vibration detection under certain conditions. The distributed detection of local temperature gradients by evaluating C-OTDR intensity changes was previously proposed by Garcia-Ruiz et al. [59,60], though their method required averaging in the time and space domains due to fading and temperature changes were not accurately quantified. In this paper, we show that absolute temperature changes can be quantitatively determined in real time by observing the corresponding, comparatively slow sinusoidal intensity modulation at discrete sensor positions in the middle of the aforementioned sensing zones, given that monotonous temperature change can be assumed during the time interval of interest and that there are no mechanical perturbations in the corresponding frequency range. For complementary cases, we show that low-frequency vibrations down to sub-Hertz frequencies can be detected, which is not possible using standard SMF and C-ODTR, if environmental temperatures remain reasonably stable. We also demonstrate that the fs-inscribed dots can be used to enhance the local backscatter ampitude specifically at long sensing distances, thus recovering local sensitivity to dynamic strain.

It is our intention to demonstrate, in a comprehensive way, the sensory characteristics and benefits of this scattering dot-enhanced amplitude-based C-OTDR DAS/DVS sensing scheme. This cost-effective scheme combines simplicity and robustness on the interrogation side with that on the sensing fiber side. Nevertheless, we were able to gain attractive sensing performance results. This makes the proposed scheme, in our opinion, a viable technique for commercial use, especially where large sensing ranges are required. An example for a suitable and highly relevant field of application, and one major motivation for this work, is DVS-based condition monitoring of submarine power cables [61,62,63,64].

This paper is organized as follows: in Section 2, we briefly introduce the theoretical background and the main conceptual ideas of the sensing method, in Section 3 the backscatter characteristics of the scattering dots are discussed, in Section 4 the regularization of the transfer function is demonstrated, in Section 5, we show, via experimental results, that simultaneous quantitative temperature gradient sensing and distributed vibration sensing is feasible for certain monitoring applications, and in Section 6 we demonstrate via experimental evidence that the presented method allows for sub-Hertz vibration demodulation, that sensitivity fading is in fact mitigated and that sensitivity can be boosted via the backscatter enhancement from the dots at large sensing distances, respectively. We summarize our results in Section 7.

## 2. Theory and Concept

Rayleigh C-OTDR, in general, is a distributed interferometric method and involves a coherent optical pulse of width *W* propagating through the sensing fiber and inducing Rayleigh scattering from commonly occuring randomly distributed refractive index (or density) flucuations. Due to the pulse coherence, the Rayleigh backscatter waves originating from the many scatterers in a fiber segment which is currently being irradiated by the pulse and thus forming the respective current resolution cell, have a defined phase relationship. The interference of the many individual backscatter waves result in a speckle-like pattern from this composite interferometer depending on the phase differences of all pairs of backscattered waves [9,11,14,59]. These phase differences are each highly sensitive to mechanical and thermal influences, resulting in significant flucuations of the effective backscatter intensity in the time and the spatial domain, respectively. This, in turn, leads to a flucuating local sensitivity, the well-known fading phenomenon [12,13,14]. The randomness of the scatterer distribution and thus the distribution of relative phases leads also to a quasi-random nonlinear transfer function. This nonlinearity is the origin of the fact, that with using traditional direct detection single frequency C-OTDR for distributed sensing, signals can only be detected but not quantified with respect to their amplitudes.

### 2.1. C-OTDR and Standard Optical Fiber

With standard C-OTDR and common optical fiber, the output intensity I(z) for a fiber position *z* results from a complex interference pattern originating from the interference of many Rayleigh backscatter waves reflected from *M* randomly distributed scatterers within a fiber segment of length W/2 that is currently irradiated by the coherent interrogator pulse [11,22]. All phase differences $\left(\phi_{i}-\phi_{j}\right)$ between each pair of partial backscatter waves i,j within the pulse width affect, via a cosine term which is weighted with the reflectivities $r_{i},r_{j}$ of the involved scatterers and the initial intensity $I_{0}$, the output signal of the local effective composite interferometer [9,11,14,22,28]:

(1) $$I\left(z\right)\propto I_{0}\sum_{m=1}^{M}r_{m}^{2}+2I_{0}\sum_{i=1}^{M-1}\sum_{j=i+1}^{M}r_{i}r_{j}\cos\left(\phi_{i}-\phi_{j}\right)$$

The first term is due to incoherent backscatter and is not much affected by external signals. The coherent second term, however, varies with external stimuli as the relative phases are affected by mechanical and/or thermal inputs. The significant nonlinearity of standard C-OTDR becomes apparent looking at the second term since the relative phases $\phi_{i,j}$ of a large number of relevant scatterers are susceptible to stimuli; the entirety of which in turn determine the component of the output signal intensity I(z) that is affected by perturbations.

### 2.2. C-OTDR and Structured Optical Fiber

**Figure 1:**
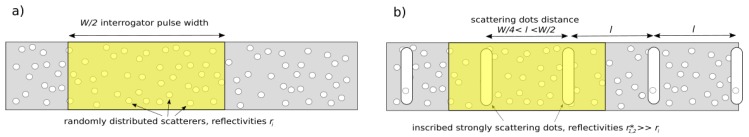
Sketch illustrating the difference in arrangements between an unmodified standard optical fiber and a modified scattering dot fiber using standard coherent optical time domain reflectometry (C-OTDR). (**a**) unmodified standard fiber, (**b**) scattering dot fiber.

Let us now compare the above case with one where the sensing fiber is structured with equally-spaced strongly scattering dots. This mirrors the approach in [52] where equally-spaced UV dots were used. The distance between neighboring dots *l* is chosen such that, within the selected interrogator pulse width W/2, there are exactly two strong scatterers, i.e., W/4<l<W/2 [20,28,32,34]. These two dots have the individual phases $\phi_{1}^{*},\phi_{2}^{*}$ and reflectivities $r_{1}^{*},r_{2}^{*}\gg r_{i,j}$. The arrangment in comparison to unmodified optical fiber is illustrated in the sketch in Figure 1.

The strong backscatter from the inscribed dots overwrite the local nonlinear transfer function as the output of the local composite interferometers is now dominated by the interference between the backscatter waves from the two dots. Equation (1) can thus then be simplified to [28,29,32]

(2) $$I\left(z\right)\propto I_{0}\left({\left(r_{1}^{*}\right)}^{2}+{\left(r_{2}^{*}\right)}^{2}\right)+2I_{0}r_{1}^{*}r_{2}^{*}\cos\left(\Phi \right)$$

The phase difference $\Phi =\phi_{1}^{*}-\phi_{2}^{*}$ is then the phase of a two-beam interferometer, where the scattering dots take the role of reflectors. This phase (or the optical path length between the dots) is very sensitive to perturbations. In the reduced form in Equation (2), the output intensity I(z) varies with a phase change from a given phase position Φ of the single interferometer via a cosine. Structuring the fiber with equally-spaced scattering dots thus makes the approach a quasi-distributed Fabry-Perot interferometric one.

Equation (2) shows that the measured signal is only to a much lesser degree affected by thermal noise as compared to the complex composite random phase relation descibed by Equation (1). Since the integral phase change over the distance of the scattering dots is much less susceptible to thermal noise, sensitivity fading is thus mitigated. The phase Φ is given by [11,29,65,66]

(3) $$\Phi =\frac{4\pi nl}{\lambda }$$

where *n* is the refractive index of the medium between the refĺectors (scattering dots), *l* is the distance between them, and λ is the wavelength of the interrogator pulse. Φ depends on the refractive index *n* and the distance *l*, both of which are affected by external stimuli, be it thermal or mechanical.

### 2.3. Change of Local Interferometer Phase with Temperature Variation

In the case of a temperature change, for example, Φ changes due to the thermo-optic effect and thermal expansion of the relevant fiber segment [66]:

(4) $$\frac{\partial \Phi }{\partial T}=\frac{4\pi l}{\lambda }\frac{\partial n}{\partial T}+\frac{4\pi n}{\lambda }\frac{\partial l}{\partial T}=\frac{4\pi l}{\lambda }\left(\frac{\partial n}{\partial T}+\alpha n\right)$$

where $\frac{\partial n}{\partial T}$ is the thermo-optic coefficient and $\alpha =\frac{\partial l}{\partial T}$ is the thermal expansion coefficient of the fiber material. With a temperature change ΔT, the resulting change of phase ΔΦ can with Equation (4) be written as

(5) $$\Delta \Phi =\frac{4\pi l}{\lambda }\left(\frac{\partial n}{\partial T}+\alpha n\right)\Delta T.$$

There is thus a linear relationship between temperature change ΔT and phase change ΔΦ of the local interferometer formed by the pair of scattering dots.

Due to the cosine nature of the phase dependence of the intensity (Equation (2)), an approximate phase change over time resulting from a monotonous temperature change can then be estimated from the traversed periods in the modulated output intensity time series. Therefore, the (absolute) temperature change can be quantitatively determined in real time with a corresponding resolution as determined by the parameters in Equation (3). The proposed method thus allows for (quasi-)distributed temperature gradient sensing (DTGS) with a spatial resolution determined by the spacing of the scatterers (and the appropriately chosen interrogator pulse width). A limiting factor is the sampling rate, so that phase changes can be monitored sufficiently. However, since realistic temperature changes for most monitoring purposes lead to phase changes below a few Hz, this should not limit the applicability of the proposed method for even long range monitoring applications [59,60] since the sampling frequency is in the kHz region. One important exception would be fiber optic fire detection, which can involve high frequency signals due to the large temperature gradients. Note that this method only allows for the determination of absolute temperature change as the sign of ΔΦ and thus the polarity of the optical path length change can not be determined by observing the output of an amplitude-based single wavelength C-OTDR. The applicability of the proposed method therefore is limited to cases where monotonous temperature changes can be assumed for distinct time intervals (e.g., developing hotspots in cable installations). The sign of temperature change could be determined using other techniques. In previous work, where the fiber was structured with FBG arrays, the issue of indeterminable signal sign was treated by using multiple interrogator pulse wavelengths [29,30,31,33], which enabled actual phase recovery and thus determination of signed changes also for time-varying perturbations.

## 3. Characteristics of Scattering Dots

The purpose of the scattering dots is to provide very localized Rayleigh backscatter of definable spacing along the fiber which is strong enough to dominate the backscatter from all other randomly distributed scattering centers in the fiber core within a selected C-OTDR interrogator pulse width yet not strong enough to introduce unacceptable attenuation and thus severely reducing the sensing range. Each scattering dot used for our experiments was inscribed in the fiber core of standard SMF using a single fs-pulse. The fiber modification was carried out by the Fraunhofer Heinrich Hertz Institute (HHI) Goslar, Germany using the method described in [57,58].

**Figure 2:**
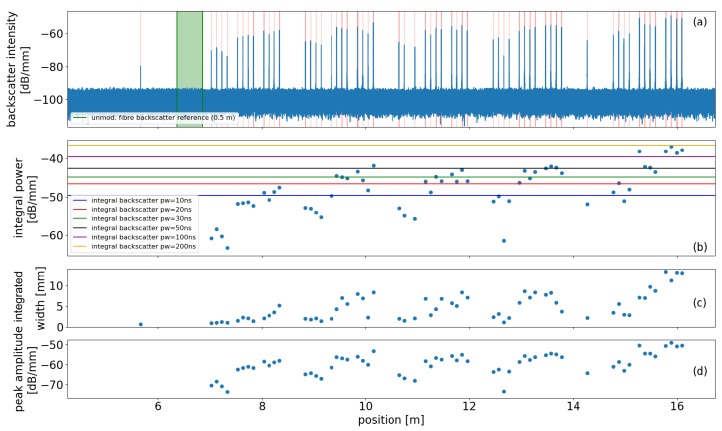
Properties of scattering dot specimen inscribed into the core of standard single mode optical fiber for different inscription parameters. (**a**) Distributed backscatter intensity; the green shaded (unmodified) segment is used as backscatter reference. (**b**) integral power of scattering dots; the horizontal lines depict the integral backscatter power levels of segments of unmodified standard fiber for various interrogator pulse widths *W*. (**c**) Integral width of peak as determined by OFDR and (**d**) peak amplitude of individual scattering dots.

Figure 2 shows an analysis of an SMF (Corning) test specimen with inscribed scattering centers produced using fs-pulses under different process parameters. Panel (a) shows the distributed backscatter intensity as determined by a commercial Rayleigh OFDR device (Luna OBR 4600, Luna Inc., Roanoke, VA, USA), (b) shows the integral backscatter power of the detected backscatter peaks, panel (c) shows the corresponding virtual widths of the scatterers used for power integration and (d) displays the peak backscatter amplitudes of each scatterer.

The analysis plot in Figure 2 shows 60 inscribed scatterers with varying inscription parameters, grouped in five times three times four scatterers (outer to inner). The varied parameters for these subgroups are (from left to right and outer to inner): 5 different inscription pulse energies 100 nJ, 150 nJ, 200 nJ, 300 nJ, 400 nJ; 3 different inscription focus points: radial offset from core center 4 μm, radial offset from core center 2 μm and core-centered; 4 scatterers with same process parameters.

**Figure 3:**
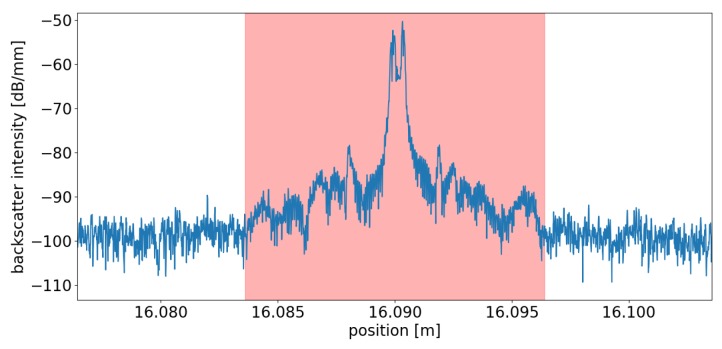
Detailed view of single scattering center. The shaded area depicts the (virtual) integration width from which the scatterer’s integral power is calculated.

The horizontal lines in panel (b) denote the integral backscatter power levels corresponding to interrogator pulse widths *W* of different durations for an unmodified SMF calculated from the green shaded reference segment shown in panel (a). This means, e.g., that the rightmost four scatterers exhibit (together as pairs) an integral backscatter power larger than the resulting integral incoherent backscatter amplitude from an interrogation length of up to 100 ns of a plain SMF fiber. Consequently the backscatter amplitude of each pair of fs-dots dominates the backscatter amplitude for coherent detection as well. A detailed view of a single scatterer can be seen in Figure 3. The shaded area signifies the virtual width of the scatterer from which the scatterer’s integral power is determined.

Each dot was inscribed using a single fs pulse with a spot size <1 μm [58]. The rather large apparent size of the dot as well as its double-peak structure as depicted in Figure 3 stems from the Dirac delta-like increase of backscatter amplitude induced by fs-laser, which could not be properly recovered by the inverse FFT of the OFDR device. Therefore the integral power was used for the tuning instead of the peak backscatter amplitude. The results show, that the backscatter of the inscribed dots (reflectivity) can be tuned via the inscription pulse energy to the desired level relative to the backscatter of unmodified segments of standard optical fiber.

This dot inscription is a simple yet efficient way to produce reflectors of defined distance and well-reproducible backscatter amplitude. Their manufacturing for use as high quality and consistent point reflectors is easier and more cost-effective when compared to other methods like large FBG arrays or continuous gratings. Nevertheless, until recently investigations regarding quasi-distributed interferometric DAS/DVS focused almost exclusively on FBGs. However, recently another group has described the method of femtosecond laser inscription of backscatter enhanced dots and outline the benefits [53,54,55], though so far no in-depth sensing performance analysis has been published.

## 4. Regularization of Transfer Function

In order to demonstrate the regularization of the sensing fiber transfer function, we carried out experiments subjected our sensing fiber to a monotonous temperature gradient. A change in environmental temperature should result in a corresponding slow linear change of the phase Φ according to Equation (5) which is observable in a cosine-like modulation of the measured output intensity I(z) according to Equation (2). Exposure to a prolonged monotonous temperature gradient thus means mapping the transfer function over many periods. Additionally introduced simultaneous vibrations with a constant small amplitude (with a resulting phase shift ≪2π) should result in a phase modulation within the transfer function which is detectable via the signal strength. This signal strength reflects the varying local interferometer’s phase state, the tuning of which is imposed by the temperature gradient.

### 4.1. Sensing Fiber and Experimental Setup

**Figure 4:**
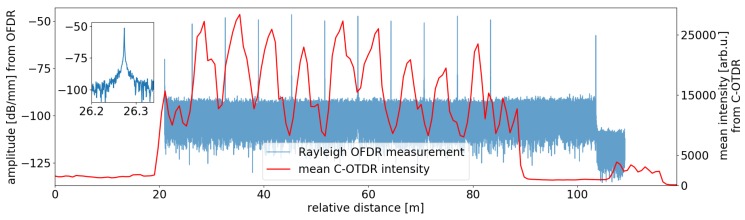
Backscatter profile of utilized sensing fiber from Luna OFDR measurement (blue line) and averaged C-OTDR intensity with a selected interrogator pulse width of 10 m. The inset shows a zoomed view upon a single scattering dot.

Figure 4 (blue line) shows the backscatter profile of the sensing fiber used for the experiments as measured by a Luna OFDR. The fiber has 10 inscribed scattering dots equally spaced by 6.48 m. The red curve shows a long-term average of C-OTDR intensity traces using an interrogator pulse width of 10 m (pulse duration 100 ns). The chosen pulse width correspondingly results in an interference pattern with 9 peaks with each maximum located in the middle of an encompassing pair of scattering dots. The inset shows a single scattering dot.

**Figure 5:**
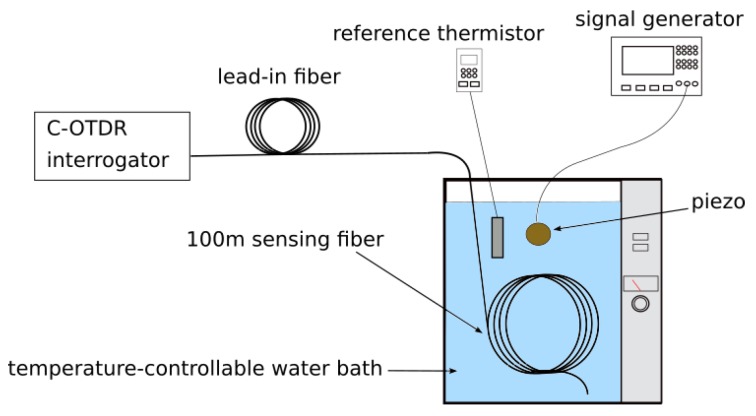
Experimental setup with scatterer dot sensing fiber in temperature-controllable water bath. Vibration is employed via a submerged piezo operated via a signal generator. The temperature evolution was measured with an Almemo thermistor for reference.

Figure 5 shows the experimental setup. The sensing fiber was connected via a lead-in fiber to a commercial DAS/ DVS-interrogator device (Helios HSI, Fotech Solutions Ltd., Hampshire, UK) which is an amplitude-based single wavelength C-OTDR (without phase recovery capabilities). The modified fiber section was placed in a temperature-controllable water bath, with the varying temperature being recorded by a reference Pt100 based thermistor (Ahlborn GmbH, Hildesheim, Germany). Also submerged in the water was a piezo electric buzzer controlled by a signal generator.

During the course of the experiment, the water temperature was increased from T=24.7 °C to T=31.2 °C over a time period of approx. 10 min., meaning a cumulative temperature change of ΔT=6.5 °C. Meanwhile, the piezo permanently induced vibrations with frequency f=700 Hz in the water to be detected by the DVS device while the local backscatter intensity varied due to the temperature gradient.

### 4.2. Results

**Figure 6:**
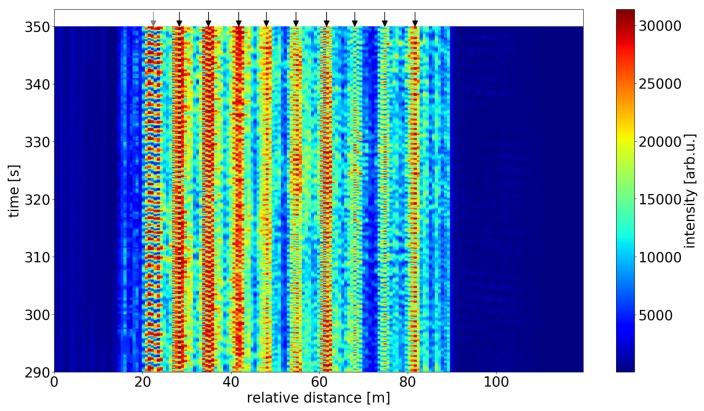
C-OTDR raw data along scatterer sensing fiber during heating. The black arrows mark the sensor positions used for sensing. The local patterns result from backscatter interference from pairs of consecutive scattering dots and are highly sensitive to thermal and/or mechanical excitation. The signal shown at the position indicated by the grey arrow stems from interference between the reflections from a lead-in fiber splice and the first scattering dot, respectively.

Figure 6 shows the distributed C-OTDR raw data along the scattering dot sensing fiber during a 1 min interval. The dynamics show a clear periodic modulation of the signal under the influence of the applied heat in 9 distinguishable interference maxima (9 pairs of 10 subsequent dots), as marked by the black arrows. The leftmost modulations (marked by grey arrow) stem from interference between the reflections from the leftmost scattering dot and the lead-in fiber splice.

**Figure 7:**
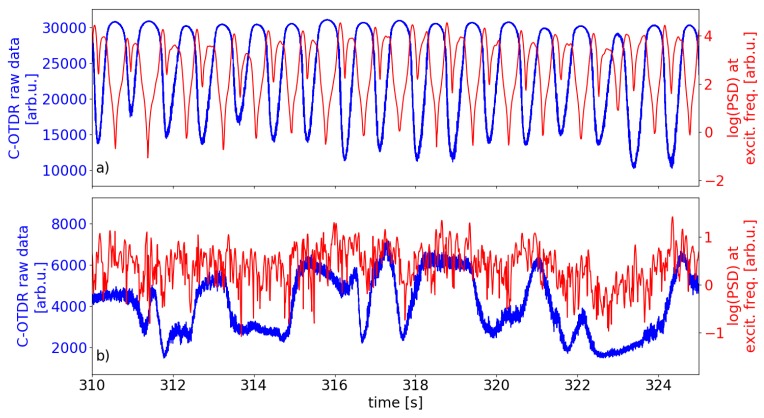
Intensity dynamics and current signal strength during ongoing heating process: timeseries of C-OTDR raw data (blue) in single sensor bin and corresponding log PSD at excitation frequency f=700 Hz (red) over time. (**a**) sensor bin at sensitivity maximum within modified segment of fiber (**b**) sensor bin in unmodified segment of fiber.

A zoomed view of the raw signal from a single sensor position located at one of the sensitivity maxima is depicted in Figure 7a (blue line). In agreement with our hypothesis, the C-OTDR trace indeed shows a sinusoidal-like behavior. In that way, it behaves like a two-beam interferometer whose phase is tuned continuously in one direction. The shown dynamics demonstrate that the transfer function is regular instead of randomized like is the case for unmodified standard SMF. Subsequent maxima mark a full 2π change of the (return) phase Φ of the local interferometer made up of the encompassing pair of scattering dots.

For comparison with the case of plain fiber, Figure 7b (blue line) shows the dynamics in a single sensor range bin within the same time interval as depicted in (a) within a submerged yet unmodified segment of the sensor fiber. In contrast to the regular sinusoidal modulation of the intensity signal in the middle of the sensing zones in the structured part of the sensing fiber, the dynamics here behave erratically owing to their intrinsically random transfer function. From the depicted waveform it is impossible to make a statement about the current sensitivity or the temperature gradient for that matter.

Now we want to assertain how the regular transfer function translates to sensitivity of small amplitude vibrations. As mentioned above, during the heating process the submerged piezo device permanently induced vibrations of constant amplitude in the water bath which the submerged sensing fiber was detecting. The current interferometer phase state, and thus its operating point within the transfer function was continuously tuned due to the optical path length of the interferometer increasing with the temperature increase. The modulation due to the vibrations depends on the current operating point within the transfer function and manifests itself in a varying signal strength. To quantify the varying signal strength over time, we choose the power spectral density (PSD) at the piezo frequency f=700 Hz within short time intervals. The red line in Figure 7a depicts the log of this time-dependent PSD as determined from short-time Fourier transform (STFT) spectra of subsequent windows of length 0.1 s with an overlap of 0.09 s. As expected, the signal strength exhibits minima when the current output intensity is at or close to local extrema and shows maxima meaning maximal sensitivity to vibrations during the linear segments of the intensity dynamics, following the derivative of the backscatter signal. This indicates that the DVS sensitivity in fact depends on the phase state (operating point) of the local interferometer formed by a pair of the inscribed scatterers which itself has a regular transfer function. Our measurement results are indications for a linear phase response to vibrations, however, our experiments are unsuitable to validate that hypothesis. Nevertheless, in cases where the transfer function has been mapped like in our experiment and the current interferometer state can thus be estimated and is stable, the amplitude of a small signal vibrational perturbation can rougly be gauged from the resulting intensity modulation. In the case of stronger signals of known frequency, resulting in an of overmodulation of the local interferometer, the excitation magnitude could possibly be estimated by determining the most dominant higher harmonic frequency, which would not be feasible with a random transfer function. This question, however, still needs to be properly evaluated.

As expected, the corresponding signal strength for the sensor bin in the plain fiber segment behaves erratically due to the latter’s random transfer function.

## 5. Simultaneous DVS and DTGS

Because the inscription of the strongly scattering dots regularizes the transfer function, we can quantify unidirectional phase shifts on the scale of full periods (i.e., multiples of 2π) within a certain time interval. It is thus feasible to utilize the C-OTDR traces from the sensitivity maxima in the middle of each inscribed scatterer pair for simultaneous distributed temperature gradient sensing (DTGS) and distributed vibration sensing (DVS) if certain conditions are met.

In many monitoring applications, it is of interest to measure moderate temperature gradients while simultaneously detecting vibrations occuring at the same time. These kinds of temperature gradients result in phase shifts that occur on much slower time scales than common vibration frequencies. The influence of vibrations and temperature drifts can this way be separated spectrally. This is of course only feasible in applications where no low-frequency vibrations or transient strain, respectively, are expected. If, on the other hand, sensing targets low-frequency vibrations (like demonstrated in Section 6.3), simultaneous DVS and DTGS using the scheme proposed here is not possible because signal frequencies could overlap and the effects therefore not be separated. Analogous, high temperature gradients can occur in some important monitoring applications as well, like e.g., fire detection. Fast (monotonous) temperature changes lead to large phase shifts in a short time. The resulting higher frequency signal can then in general not be distinguished from vibrations and our scheme thus not be employed.

Our proposed method for simultaneous DVS and DTGS in a single fiber depends on the requirement that the strong phase modulation (by temperatur or strain) take place on slower time scales than the vibrations that are to be monitored. The current temperature gradients can then be determined from the slowly varying component of the measurement data and for vibration sensing the higher frequency bands can be used since vibrations are modeled as elastic processes. Previously published work demonstrated that local temperature gradients could be detected from analyzing the intensity output of traditional C-OTDR [59,60]. However, averaging in the time and spatial domain was necessary due to the randomly distributed scatterers in the used standard optical fibers and the ensuing intensity fluctuations; the temperature gradients could also not be precisely quantified. In the present case, because of our fiber modification, the transfer function is regularized and smooth intensity modulations occur. These modulations translate from monotonous phase shifts directly proportional to introduced temperature variations and can be observed at fixed positions.

For the experiment described in Section 4.1, we now want to compare the temperature values as measured by the reference thermistor with those calculated from Equation (5) using the C-OTDR dynamics in a single sensor position for the observable phase change ΔΦ. As mentioned earlier, a modulation of the dynamics from peak to peak means a phase shift of ΔΦ=2π, since the return-trip phase difference between two paired scattering dots needs to be considered. From Equation (5) it then follows with a spacing l=6.48 m between the scattering dots, the C-OTDR interrogator wavelength λ=1540.55 nm, the thermo-optic coefficient of fused silica (the sensing fiber core material) $\frac{\partial n}{\partial T}=8.57\times 10^{-6}$ K${}^{-1}$ [67,68], the thermal expansion coefficent of fused silica $\alpha =0.55\times 10^{-6}$ K${}^{-1}$ [68] and the refractive index of SMF-28 of n=1.468 that a temperature variation of ΔT=1 °C results in a phase shift of $\Delta \Phi_{1{}^{\circ}C}=156.8\pi$ or alternatively that an observable phase shift of ΔΦ=2π (peak-to-peak in the C-OTDR trace) means an absolute temperature change of $\Delta T_{2\pi }=0.0127$ °C. This implies a very high temperature gradient resolution of our method.

**Figure 8:**
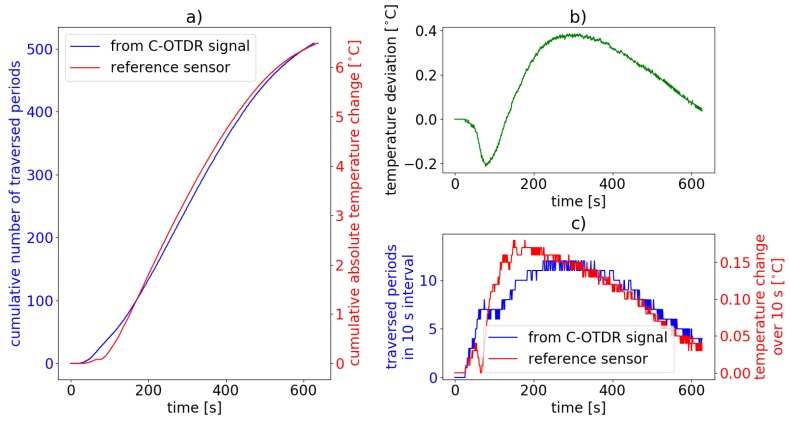
Distributed temperature gradient sensing result for single sensor position during heating process. (**a**) cumulative number of traversed periods from the C-OTDR trace (blue) and temperature as measured by the reference thermistor (red), (**b**) deviation between measured relative temperature increase and temperature increase as calculated from Equation (6), (**c**) corresponding number of traversed periods (blue) and simultaneously measured temperature changes (red), both over 10 s intervals.

For the evaluation of our approach for DTGS we simply count the number of peaks $N_{p}\left(t\right)$ in the raw data trace within a fixed time interval of 10 s. $N_{p}\left(t\right)$ corresponds to the number of full traversed periods, i.e., a phase shift of $\Delta \Phi \left(t\right)=N_{p}\left(t\right)\cdot 2\pi$. The time-dependent temperature gradient can then be calculated from

(6) $$\Delta T=\Delta T_{2\pi }\cdot N_{p}\left(t\right),$$

or the total temperature change from the number of all peaks in the C-OTDR data. The results are displayed in Figure 8.

Figure 8a shows the evolution of the cumulative number of full periods traversed in the C-OTDR trace (blue line) and the relative temperature increase as measured by the reference thermistor (red line). The plot in (b) shows the deviation between the absolute temperature change as calculated by Equation (6) with the number of periods $N_{p}$ and the measured relative temperature change measured by the thermistor. Figure 8c depicts the corresponding number of periods and the measured temperature changes in 10 s windows, respectively (same colors). It is apparent that the result from C-OTDR based DTGS agrees quite well with the reference sensor. Even though there are temporary deviations of up to 0.4 °C, the determined temperatures at the end of the measurement differ by only 0.038 °C. The curves shown in Figure 8c exhibit a high level of congruence. This implies a linear response of the sensor to introduced temperature changes, at least on the 10 s time scale and a phase change resolution of subsequent full periods.

We assume that large parts of the intermittent deviation between the measurements of our distributed fiber optic sensing method and of the conventional point sensor, respectively, originate from insufficiently isotropic heat distribution in the water bath. Furthermore, the heat capacity, thermal conduction and sampling, respectively, for both sensors do not match perfectly, which can cause a temporal offset for the temperature retrieval.

We have thus shown, that our method allows for simultaneous real time DTGS and DVS with traditional C-OTDR in a single fiber in cases with low to moderate temperature gradients.

## 6. DVS Performance Benefits of Using Scattering Dots fFber

### 6.1. Motivation Power Cable Monitoring

When using the modified structured sensing fiber for C-OTDR DVS, several other performance improvements as compared with cases where standard SMF is used arise. Here, we demonstrate via experimental results that sub-Hertz vibrations can be resolved, that fading is mitigated and that sensitivity can be boosted, especially at long sensing distances. Our experimental setup involves optical fiber embedded in a power cable which was originally intended for telecommunications purposes. This test object was chosen because our motivation is the application of our method for vibration (and temperature) monitoring of extended power cable installations, e.g., subsea power cables connecting offshore wind farms with the shore [61]. For this application, a long sensing range is key [55,62,64], but also the ability to demodulate and localize low frequency vibrations, e.g., from anchors being dragged over the seabed close to and/or towards the submarine cable, is highly desirable for transmission system operators (TSO) to avert damages or failures [62]. Furthermore, determining dynamic or transient strain values in the power cable for conditions monitoring during the power cable installation process via DVS [63] is an attractive application that could allow for a early detection of damages to the cable sustained during laying thus saving costs and preventing later occuring secondary damages.

### 6.2. Experimental Setup

**Figure 9:**
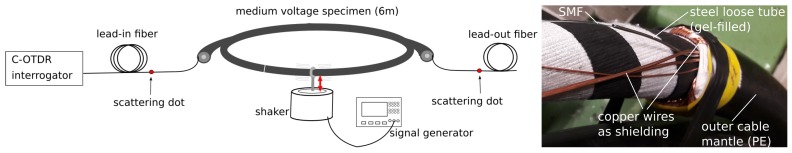
Left: setup for vibration sensing in a medium voltage cable. One scattering dot was spliced into each the lead-in fiber and the lead-out fiber, respectively, resulting in a distance between them of 6.7 m. Right: detailed view of one end of the power cable with single mode optical fiber (SMF) in stainless steel loose tube embedded in the shielding layer made up of copper wires.

Our laboratory setup is depicted on the left of Figure 9. The test object is a 6 m long coiled medium voltage power cable specimen with a standard SMF in a gel-filled steel tube embedded in the outer shielding layer made up from copper wires. A photo showing details of the cable is depicted on the rhs of Figure 9. The embedded fiber was on one side connected via a lead-in fiber to our commercial C-OTDR interrogator (Helios HSI). On the other end, the embedded fiber was spliced with a lead-out fiber.

On each side of the power cable a short fiber segment with one of the above described scattering dots was spliced with the lead-in and the lead-out fiber, respectively, resulting in a single pair of dots with a fiber distance between them of 6.7 m. The scattering dots on either side of the power cable together with 30 m of the lead-in or the lead-out fiber, were each boxed and cushioned by foam to exclude vibrations not exerted on the power cable from influencing the measurements. The power cable was attached to a lab shaker via a specially made clamp. The shaker vibrations were controlled by a signal generator. The power cable and the lead-in and lead-out fibers were positioned such that the vibrations would not result in significant longitudinal dynamic strain between the optical fiber coming out of the ends of the power cable and the lead-in our lead-out fibers, respectively.

This setup was used for all experiments in the following subsections and allowed us to introduce vibrations of arbitrary frequency to the power cable to be sensed by the fiber segment between the scattering dots. By varying the length of the lead-in fiber (55 km for the experiments described in Section 6.3 and Section 6.4, 80 km for the experiment in Section 6.5) we could ascertain the performance of the single dot pair sensor at different sensing distances. To be able to profit from the scattering dot sensing scheme, the distance between the introduced dots required us to set the C-OTDR interrogator pulse width to 10 m (100 ns pulse duration) during the experiments described in this section.

### 6.3. Resolution of Sub-Hertz Vibrations

One sensory advantage of using the modified sensing fiber with inscribed scatterers is the possibility to resolve low-frequency vibration signals. This was very briefly demonstrated in previous work [51] and analogously for discrete strong scattering segments produced by UV exposure [52], each under ideal lab conditions. Here we present results using a setup closer to reality, i.e., without thermal insulation and with power cable monitoring close to the real application.

When using standard optical fiber to detect low-frequency vibrations, unavoidable thermal drifts together with the commonly random transfer function result in a sustained noise-like background in the low frequency band. In usual conditions, vibrations with frequencies f<5 Hz are thus unresolvable.

However, we demonstrated that the structuring of our sensing fiber with inscribed scattering dots and the resulting regularization of the transfer function (as shown in Section 4) enables the demodulation of low-frequency (sub-Hertz) signals. If relatively stable thermal conditions can be assumed, corresponding signal components can be considered negligable. Thermal drifts of course still persist, but due to the significant integration length of the relevant phase shift ΔΦ of the order of meters, the transfer function can be considered stable. Low frequency vibrations exerted on the sensing fiber should then be detectable. It should be noted, that these requirements naturally exclude the possibility of simultaneous DVS and DTGS as described in Section 5 for applications where low frequency vibrations need to be detected or monitored as our scheme depends on spectral separability of signals induced by vibration and thermal influences, respectively. In order to demonstrate sub-Hz signal detection, we introduced low frequency vibrations with a frequency f=0.6 Hz via the shaker. The sensing distance of the relevant fiber segment bounded by the scattering dots was approx. 55 km. To this end, a lead-in fiber made up of 25 km Corning Ultra Low Loss (ULL) fiber and 30 km standard SMF (j-fiber, Jena, Germany) was used. The sampling rate (pulse repetition frequency) was 1 kHz.

**Figure 10:**
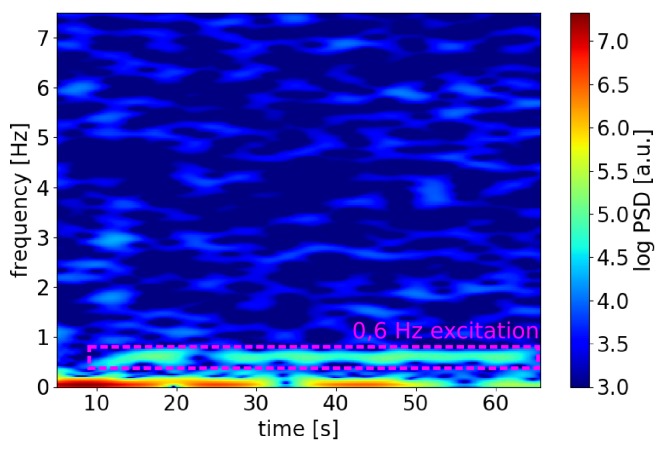
Spectrogram calculated from C-OTDR signal in single sensor segment within the power cable at a sensing distance of more than 55 km . The excitation frequency f=0.6 Hz is clearly visible.

Figure 10 shows a spectrogram calculated from the backscatter signal measured at a single sensor position within the power cable specimen. The spectrogram was produced with an FTT window length of 10 s and a step size of 0.1 s. The excitation frequency of 0.6 Hz is clearly visible, the frequency components at even lower frequencies we attribute to not completely stable environmental temperatures. We therefore were able to demonstrate that sub-Hertz signals originating from dynamic strain, too, can be resolved using our method.

### 6.4. Fading Mitigation

**Figure 11:**
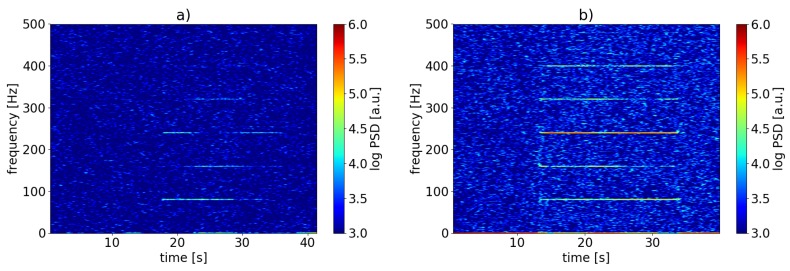
Spectrograms calculated from C-OTDR signals in single sensor segments within power cable with applied vibration with f=80 Hz at a measurement distance of 55 km. (**a**) for unmodified standard SMF; (**b**) for the case using fiber-inscribed scattering dots.

Another benefit of our scheme is the stabilization of the sensitivity meaning the well-known fading phenomenon which is typically associated with C-OTDR [12,13,14] is mitigated. Due to the normally random transfer function of standard optical fiber, sensitivity usually fluctuates over time. This implies that within a resolution cell as defined by the propagating interrogator pulse width, sensitivity fluctuates also in the spatial domain. Substituting the random transfer function with that of a simple two-reflector interferometer of a length comparable to that of the resolution cell results in less susceptibility to noise-like thermal drifts. We validate this via another set of experiments. During the first measurement, we used the above-described experimental setup, but without any spliced-in scattering dots framing the power cable. During the second measurement, the setup was as shown in Figure 9. This is meant to illustrate the performance benefit comparing the cases with scattering dots and without them. The lead-in fiber had a length of approx. 55 km and was composed like described in the previous subsection. The sampling rate was set to 1 kHz. The shaker was exerting vibrations of frequency f=80 Hz onto the power cable during the measurements. Resulting spectrograms from single sensor positions within the power cable for both measurements are shown in Figure 11. The FFT window size was 1 s with an overlap between subsequent windows of 0.9 s. Both plots show the base frequency and several higher harmonics. This common harmonic distortion results from overmodulation respectively a large phase change (>2π). Due to the stable PSD values for the scattering dot fiber, the total phase modulation could theoretically be estimated by determining the dominant harmonic frequency, since the base frequency is known. In summary, it is apparent, that without the sensing fiber modification, the PSD at the main frequencies composing the signal fluctuate or drift over time (Figure 11a), while the results for the case with employed scatterers show almost stable PSD values.

In order to attain a time-varying measure of signal strength we now consider the sum s(t) of the PSD values shown in Figure 11 at the signal frequencies f=80,160,240,320,400,480 Hz:

(7) $$s\left(t\right)=\sum_{f=n\cdot 80\;\mathrm{Hz}}\mathrm{PSD}\left(f,t\right).$$

For cross-comparability between the two fiber configurations, we define the relative normalized PSD as s(t) relative to its average over a time interval $t_{\mathrm{off}}$ at the beginning of the measurement without the vibrations $\langle s(t_{\mathrm{off}})\rangle$:

(8) $$\mathrm{relative}\;\mathrm{normalized}\;\mathrm{PSD}=\frac{s(t)}{\langle s(t_{\mathrm{off}})\rangle }.$$

**Figure 12:**
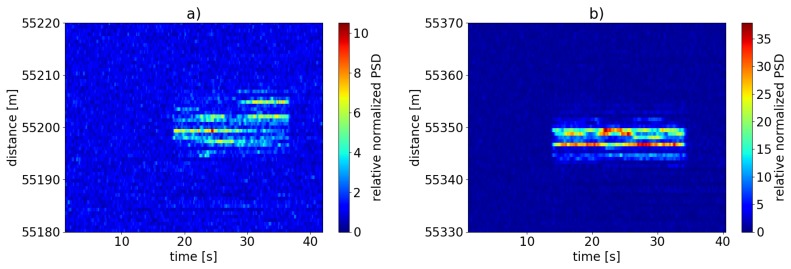
Normalized sum of power spectral density (PSD) at excitation frequency f=80 Hz and its harmonics (Equation (8)) at a measurement distance of 55 km on the basis of the spectral data shown in Figure 11a) for unmodified standard SMF; (**b**) for the case using fiber-inscribed scattering dots. The increase in sensitivity and the difference in fading behavior is clearly visible. Note the different scales of the color coding.

Figure 12 shows the evolution of the relative normalized PSD according to Equation (8) for the two measurements in spatio-temporal plots.

**Figure 13:**
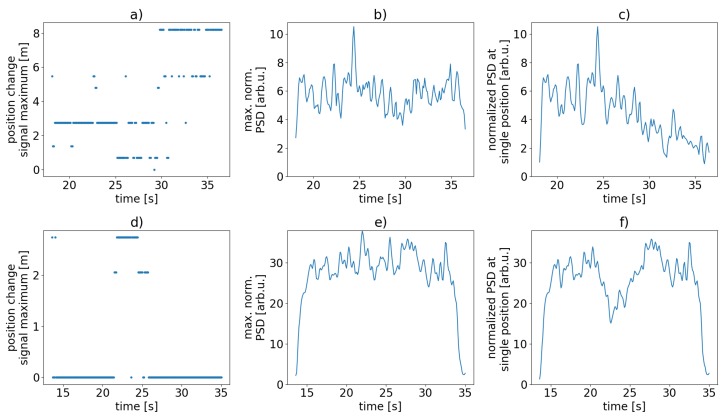
Measures showing sensitivity fading over time for the standard fiber (**a**–**c**) and for the scatterer fiber (**d**–**f**). (**a**,**d**) relative change of sensor position with the maximum signal strength (normalized PSD sum, Equation (8)); (**b**,**e**) corresponding signal strength; (**c**,**f**) signal strength in a single sensor range bin over time. Note the different scales.

The signal strength over time differs significantly between both cases: the signal strength for the case with unmodified fiber (Figure 12a) is much lower than for the scattering dots fiber (Figure 12b) and fluctuates strongly over time and space. In contrast, the results for the case with spliced-in scattering dots exhibit an almost stable spatial distribution of signal strength and much lesser fluctuations over time. The difference in fading behavior on the basis of the data shown in Figure 12 is presented in more detail in Figure 13.

The upper panels (a)–(c) depict the situation for the case without scattering dots, the lower panels (d)–(f) show the case with the dots in place. Figure 13a,d display the relative change of position of the signal strength (relative normalized PSD, Equation (8)) maxima along the FUT over time. The unmodified sensing fiber exhibits a strong spatial drift of that signal maximum while within the scattering dot FUT the signal maximum location stays the same over the entire measurement except for a short intermittent period. Panels (b) and (e) of Figure 13 show the corresponding current maximum signal strengths over time for the standard SMF and for the scattering dot FUT, respectively.

Figure 13c,f depict the signal strength over time in one fixed single sensor position within the power cable for the unmodified FUT and for the modified FUT, respectively. Figure 13f demonstrates that the signal strength in the range bin of the scattering dot sensor with the signal maximum remains more or less stable (except for short period in the middle of the measurement), while the corresponding signal strength in the unmodified sensor (Figure 13c) drifts towards noise levels over time at the end of the measurement.

The above listed insights from experiments support our claim, that the use of a sensing fiber structuring with scattering dots significantly reduces the effect of sensitivity fading. With a more or less fixed position of the sensitivity maximum in the middle of each scattering dot interferometer, spatial averaging for signal refinement [14] is not necessary to evaluate the C-OTDR signal in the corresponding resolution cell. Concluding the results, we show that our scattering dots offer an increase and stabilized spatial localization of dynamic strain events when compared to the plain fiber under test.

### 6.5. Sensitivity Boost at Long Sensing Distances

In DAS/DVS an increased sensing range is highly desired for many applications, e.g., for subsea power cable monitoring [55,61,62,69]. At large distances sensitivity may be raised by increasing backscatter intensity. Currently, many works focus on enhancing the backscatter by use of continuous gratings [38,39,40]. Also experiments with range-optimized fiber configurations combining low-loss fiber with high scattering fiber have recently been published [69]. Very recently, the approach to use of fs-inscribed reflectors in ULL fiber to attain large sensing ranges via localized enhanced backscatter has been described [55]. However, DVS performance results are lacking. Here, we demonstrate that the enhanced backscatter from our scattering dots leads to an increase in local DVS sensitivity at a very long sensing distance. The local dynamic range of the sensor is also increased which leads to a better sensing performance.

**Figure 14:**
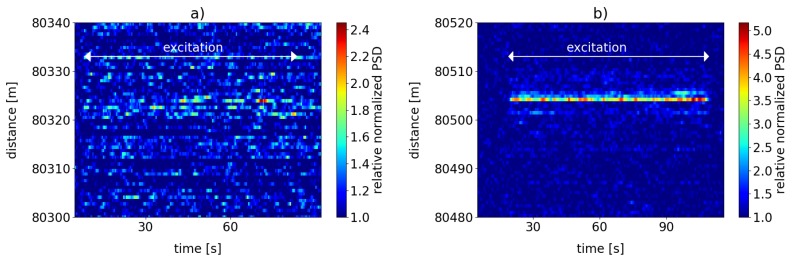
Normalized PSD sum (Equation (8)) at excitation frequency f=25 Hz and its harmonics at a measurement distance of more than 80 km. (**a**) for unmodified standard SMF; (**b**) for the modified sensing fiber with inscribed scattering dots. Note the different scales of the color coding.

At large sensing distances where available optical power is low, sensitivity can thus be recovered by the use of the scattering dots which increase the backscatter intensity. In order to demonstrate this, we performed another set of measurements analogous to those described in the previous Section 6.4 but for a sensing distance of 80 km. To that end, a lead-in fiber composed of 50 km ULL and 30 km standard SMF was utilized. The pulse repetition frequency was again set to 1 kHz. During the measurements here, the shaker introduced vibrations of frequency f=25 Hz in the power cable. Figure 14 shows the resultant signal strength (corresponding to Equation (8) but for a base frequency of 25 Hz) for the case without any scattering dots in (a) and for the scattering dot case in (b), respectively.

It is obvious, that without the backscatter inscrease by the dots, the signal can hardly be made out at all, as the signal strength levels fluctuate close to the background noise levels. In contrast, introducing the pair of scattering dot in our fiber configuration raises the signal strength and contrast significantly. The time interval of excitation can be determined with good precision, and signal levels show quite stable behavior over time.

This demonstrates that DVS sensitivity can be boosted when the modified scattering dot fiber is utilized at large sensing distances.

In order to maximize the sensing range of the presented method when using long ranges of dot-structured sensing fiber, the idea put forward in [55] to inscribe dots/ reflectors into ULL fiber should be followed. In fact, in more application-oriented research in the context of power cable monitoring we employed a DAS/DVS sensing cable manufactured on the basis of Corning ULL fiber with inscribed scattering dots intended for use over large sensing ranges. The results will be published in the future.

## 7. Conclusions/Summary

We have demonstrated the key characteristics and sensory benefits of equally-spaced fs-inscribed identical strongly scattering dots from a viewpoint of distributed fiber optic sensing based on C-OTDR. To our knowledge, this is the first time, this kind of sensing fiber structuring is investigated with full-detail, highlighting different aspects that are highly relevant for practical application.

We have demonstrated that employing a scattering dot fiber for use with conventional C-OTDR improves sensitivity to vibrations and reduces fading effects. Also, the detection of sub-Hertz vibrations is enabled. Furthermore, we could show that the use of these scatterers can enhance the possible sensing range. The feasibility to monitor and quantify monotonous temperature gradients in real time simultaneous to vibration detection in a single sensor fiber using our approach and traditional C-OTDR was also shown under certain conditions.

The regularization of the transfer function was demonstrated via mapping it via a continuously applied temperature increase. This increase could in turn be quantified due to a quasi-linear response of the sensor to temperature changes at least on the scale of full modulation periods. In this paper, we have not systematically investigated the sensor’s response to vibrations of varying amplitude in order to evaluate its linearity. This will be done in future work.

We could also not fully treat all questions that necessarily arise when fiber modifications resulting in enhanced backscatter are used, like the additionally introduced attenuation and its impact on sensing range. The limited number of scattering dots in our investigated sensing fiber resulted in no distinguishable extra attenuation. Nonetheless, our method is also meant for application over large sensing ranges. The number of scatterers necessary would be quite large if a spatially continuously structured sensing fiber of the order of tens of kilometers was to be utilized. This would result in significant losses along the fiber, though the reduced optical power at a certain sensing distance should to some degree be compensated for by the increased local backscatter. This issue will be further investigated in the future. Possible approaches to mitigate attenuation problems with structured fiber are already being investigated by several groups, like the use of special fibers, e.g., with larger numerical aperture. The scattering dots could also be inscribed in special ultra low loss fiber as was proposed in [55].

The method presented here of using dot-structured sensor fiber can in general improve the performance of DAS/DVS systems based on not only tradional C-OTDR but also on other C-OTDR schemes with more complex interrogation, detection or demodulation schemes. State-of-the-art DAS/DVS interrogators with phase demodulation capability could still benefit from the regularization of the transfer function and phase response. The mitigation of fading as well as the possibility to enhance local backscatter and this way to recover sensitivity at larger sensing distances is advantageous irrespective of the employed interrogation/ demodulation scheme.

It should be noted however, that for sensing methods based on correlation analysis of power signatures at different interrogation wavelengths like, e.g., in [22,26], such a periodic structuring with strongly scattering dots would most likely have a negative impact on sensing performance, since a corresponding efficient cross-correlation analysis relies on non-periodic backscatter profiles and benefits from the uniqueness of power signatures resulting from the random distribution of scattering centers in optical fibers.

## Abbreviations

The following abbreviations are used in this manuscript:

C-OTDR	coherent optical time domain reflectometry
DVS	distributed vibration sensing
DAS	distributed acoustic sensing
DTS	distributed temperature sensing
DTGS	distributed temperature gradient sensing
FBG	fiber Bragg grating
OFDR	optical frequency domain reflectometry
SMF	single mode fiber
FFT	fast Fourier transform
STFT	short time Fourier transform
PSD	power spectral density
ULL	ultra low loss
FUT	fiber under test
